# The Relationship Between Heart Rate and Mortality Risk in Patients With Acute Aortic Dissection: A Meta-Analysis

**DOI:** 10.31083/RCM27755

**Published:** 2025-05-27

**Authors:** Tianyi Wang, Lin Sun, Zhaozhuo Niu, Jixian Wang, Yuanshan Wang

**Affiliations:** ^1^Department of Cardiac Vascular Surgery, Qingdao Municipal Hospital, 266000 Qingdao, Shandong, China; ^2^Cardiac Surgery Unit, Qingdao Municipal Hospital, 266000 Qingdao, Shandong, China

**Keywords:** acute aortic dissection, heart rate, meta-analysis

## Abstract

**Background:**

Acute aortic dissection (AAD) is a rare but life-threatening disease, and its rapid and correct diagnosis is important. Heart rate (HR) is a risk factor for death in patients with AAD, but their relationship remains unknown. This meta-analysis aimed to evaluate whether there was a significant correlation between HR and AAD mortality risk.

**Methods:**

By searching PubMed, Embase, and Web of Science databases, the studies reporting the correlation between HR and AAD were obtained, and their methodological quality was evaluated. Relative risk (RR) with 95% confidence interval (CI) was used as the effect size. Subgroup analysis, sensitivity analysis, and publication bias test (Egger’s test and funnel chart) were used to find the source of heterogeneity and evaluate the stability of the results.

**Results:**

Ten studies enrolling >4000 patients were included. Increased HR was positively correlated with increased AAD mortality risk (RR [95% CI] = 1.04 [1.01–1.07], *p* = 0.006). There was significant statistical heterogeneity among the included studies. The timing of HR monitoring, AAD type, and follow-up time were sources of heterogeneity. Sensitivity analysis showed that the combined results were stable. There was a significant publication bias in the included studies; however, the shear-fill method showed that the publication bias had little effect on the combined results (RR [95% CI] = 1.038 [1.010–1.066], *p* = 0.008).

**Conclusions:**

There was a positive relationship between increased HR and increased AAD mortality.

## 1. Introduction

Acute aortic dissection (AAD) is a rare but highly lethal disease, with severe chest, back pain and a tearing sensation as the primary clinical manifestation [1]. AAD is usually divided into two categories; Stanford type A and B. Stanford type A involves the ascending aorta, and its treatment is primarily by surgical repair (replacement of the ascending aorta). For type A aortic coarctation, treatment can be delayed with a semi-elective surgical approach unless cardiac tamponade or malperfusion syndrome is present. Timely diagnosis and surgical treatment are important to improve the survival rate [1]. Stanford type B is based on the fact that the ascending aorta proximal to the innominate artery is not involved in the process, and its treatment is mainly medical therapy, focusing on controlling blood pressure and heart rate (HR) [2, 3]. Over time, thoracic endovascular aortic repair (TEVAR) has been recommended for complex type B AAD. However, TEVAR carries certain perioperative complications, and the timing of the treatment must be determined based on the patient’s specific condition. This typically involves monitoring the aortic diameter and assessing any new complications [4]. The comprehensive treatment for AAD has improved over the past two decades, but the mortality rates of diagnosis and treatment in hospital remain relatively high [5, 6], reaching approximately 27.4% [7].

Several factors, such as advanced age, arterial hypertension, and aortic aneurysm, are associated with adverse outcomes of AAD [8, 9]. Although the mechanisms underlying its progression remain unclear, timely and accurate diagnosis is crucial because of the exceedingly high AAD mortality rate, with an hourly death rate of 0.5%, particularly within the first 48 h after symptom onset, in which the mortality rate reaches 23.7% [1, 5]. Biomarkers, such as D-dimer [10], tenascin-C [11], and smooth muscle myosin heavy chain [12], have good diagnostic value for AAD. However, the treatment time of AAD is urgent, and the reference range of biomarkers was not completely effective in diagnosing AAD [13]. Therefore, exploring risk factors and identifying promising biomarkers to immediately identify and diagnose high-risk patients are essential.

Increased resting HR is a major risk factor for cardiovascular disease and is associated with mortality [14, 15]. Variation of HR increased the mortality of coronary heart disease, stroke, heart failure, and other cardiovascular diseases [16]. Several studies have indicated that HR is a powerful predictor of long-term mortality and may be used as a significant risk marker to predict the prognosis of patients with AAD [17, 18]. For instance, Zhou *et al*. [17] proved that HR was an independent risk factor for patients with AAD and positively correlated with long-term mortality. However, the retrospective observation in this study may cause bias, and the influence of preoperative medication on the study was overlooked. Krenz *et al*. [19] found that controlling HR through esmolol treatment could achieve the treatment of patients with AAD and evaluate the safety of this treatment method; however, this study lacked a multicenter analysis. Considering the limitations of these studies and the potential benefits of focusing on the relationship between HR and cardiovascular health for patient care [18], the association between HR and AAD should be comprehensively assessed. Here, we conducted a meta-analysis to assess HR and AAD mortality risk.

## 2. Methods

### 2.1 Literature Search and Design

The review team developed a literature search strategy in advance. Literature retrieval was performed in the PubMed, EmBase, and Web of Science databases. Search keywords included “aortic dissection”, “acute”, and “heart rate”. If the categories of keywords were similar or different, we used “OR” or “AND”, respectively, to combine them. Combined controlled vocabulary and free-text terminology was used to search the database. The specific retrieval steps of each electronic database are shown in **Supplementary Tables 1–3**. Moreover, the search language was not limited. Additionally, this study screened the relevant reviews and references of included studies to obtain more studies that could be used for meta-analysis. This meta-analysis followed the Preferred Reporting Items for Systematic Reviews and Meta-Analyses (PRISMA) guidelines.

### 2.2 Study Selection

Two researchers conducted an independent evaluation of these references. Studies were included if they met the following Population Intervention Comparison Outcome (PICO) criteria; (1) the participants were patients with AAD, and treatment methods were not limited, (2) a prospective or retrospective cohort study, (3) the study reported the relationship between HR and risk of in-hospital death and death after follow-up, and (4) the correlation strength was expressed by odd ratio (OR), relative risk (RR), or hazard ratio and 95% CI, or could be calculated according to other data. Exclusion criteria included the following; (1) case reports, editorials, and review articles, (2) non-acute patients, and (3) if multiple articles were published or had the same data, only one article was kept, including the one with the most comprehensive research information, and the remaining were excluded. If there were any differences between the two researchers, a consensual process was conducted.

### 2.3 Data Extraction and Quality Evaluation

After the documents included in the analysis were determined, the data were extracted independently according to the pre-designed table. Each included study was carefully evaluated by two independent professionals, and the extracted data included the first author, basic characteristics of the research subjects (sample size, gender, and age), HR measurement time, categories of AAD, treatment methods, and research outcomes. After data extraction, the two authors exchanged audit extraction forms and discussed and solved any inconsistencies.

According to the Newcastle Ottawa Scale (NOS) evaluation scale, the methodological quality of case-control and cohort studies was evaluated. The evaluation content included research subject selection, comparability, and exposure (eight scoring items, total score was 9) [20]. Scores of 7–9 were classified as high-quality research, 4–6 as medium, and <4 as low.

### 2.4 Statistical Analysis

For patients with AAD who reported death and survival, the study on the difference in average HR was converted into OR (95% CI) by the Chinn method [21]. In some studies, HR was used as a grouping variable, and the effect value of high vs. low HR and 95% CI were used for meta-analysis. However, some studies reported the change in risk of death for every one-beat increase in HR. Because these studies reflected the correlation between HR increase and mortality outcome, the effect values were combined. A *p* < 0.05 was considered a statistically significant difference.

RR (95% CI) was used as an effect size to evaluate the relationship between HR and AAD mortality risk. Because of the high heterogeneity of the included studies, meta-analysis was combined using the random effect model. Cochran’s Q and I^2^ tests were used for heterogeneity testing [22]. If the Q statistic *p* < 0.05 and I^2^ > 50%, the studies had statistically significant heterogeneity. If *p* ≥ 0.05 and I^2^ ≤ 50%, the statistical heterogeneity was not significant. According to other factors, such as the exposure classification, research type, region, and whether to conduct multivariate correction, subgroup analysis was performed. One-by-one elimination test was used to evaluate whether the single-inclusion study had a significant influence on the meta-analysis results [23]. By analyzing the results of Egger’s test and funnel chart, we evaluated whether the included studies had significant publication bias [24]. If there was publication bias, the influence of publication bias on the merger results was evaluated by the clipping method [25]. The above statistical analysis was completed using Stata 14.0 software (StataCorp, College Station, TX, USA).

## 3. Results

### 3.1 Included Studies and Characteristics

**Fig. 1.**
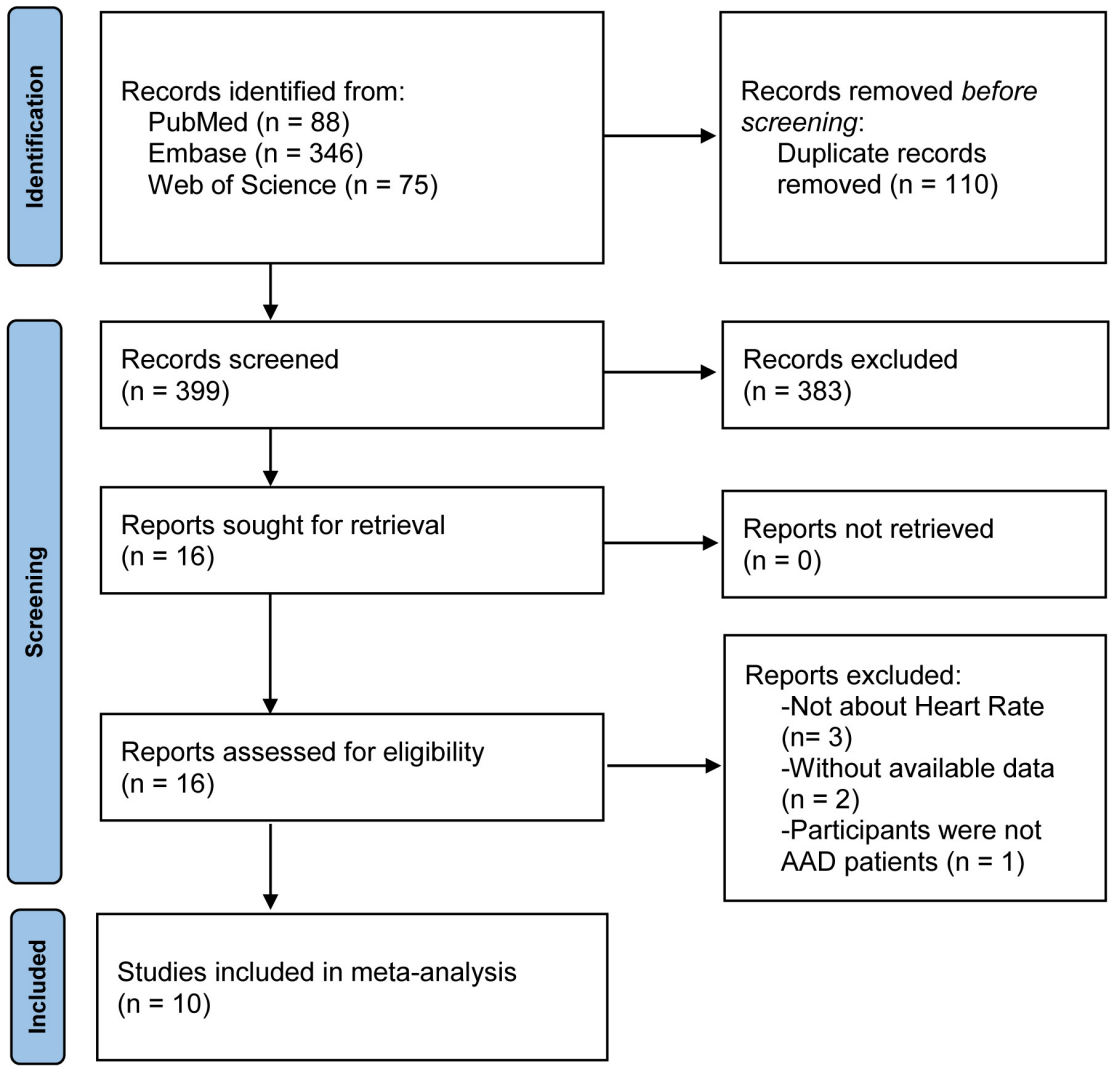
**A flow chart of the study selection process**. Abbreviations: AAD, acute aortic dissection.

This meta-analysis retrieved 509 articles in PubMed (88 articles), EmBase (346 articles), and Web of Science (75 articles). After eliminating 110 duplicate articles, 399 articles remained. After browsing the titles and abstracts, 383 articles that did not meet the inclusion criteria were excluded. Six of the 16 articles were eliminated after full-text reading, and the remaining 10 articles [17, 26, 27, 28, 29, 30, 31, 32, 33, 34] were included in the meta-analysis. The study retrieval results and screening process are shown in Fig. 1.

The characteristics of the included studies were summarized in Table 1 (Ref. [17, 26, 27, 28, 29, 30, 31, 32, 33, 34]). However, the study by Jia *et al*. [30] was a prospective cohort studies (PCS), and the other articles were retrospective cohort studies (RCS). The included studies were published from 2015–2023, and study regions were mainly distributed in China, Japan, Iran, and Israel. Among these studies, Siti *et al*. [31] reported the results of Stanford type A and B AAD. The sample size was 155–721, with 4174 individuals (2942 males and 1232 females). The average age across the studies ranged from 46.6 years to 67.5 years.

**Table 1.** **Characteristics of 10 included studies**.

Study	Location	Design	Detected time of HR	n, M/F	Age, years	Stanford type, A/B	Treatment
Chen, Z *et al*., 2023 [28]	Israel	RCS	Pre-treatment	374, 227/147	67.5 (55.3–77.4)	240/134	Medical therapy, Aortic surgery, TEVAR, ICU MV
Hagiya, K *et al*., 2021 [29]	Japan	RCS	Post-treatment	721, 368/353	65.8 ± 13.0	721/0	Surgery
Jia, Y *et al*., 2023 [30]	China	PCS	Pre-treatment	155, 125/30	55 (46–65)	96/59	Medical therapy, surgery
Ohnuma, T *et al*., 2015 [27]	Japan	RCS	Post-treatment	434, 221/213	63.3 ± 12.1	434/0	Surgery
Rahmanian, M *et al*., 2023 [26]	Iran	RCS	Pre-treatment	201, 143/58	59.9 ± 16.2	201/0	Surgery
Siti, D *et al*., 2018 [31]	China	RCS	Pre-treatment	234, 187/47	50.6 ± 12.0	88/0	Medical therapy, surgery, TEVAR
						0/146	
Wang, MM *et al*., 2023 [32]	China	RCS	Pre-treatment	715, 582/133	52.1 ± 11.8	0/715	NR
Xu, Y *et al*., 2023 [33]	China	RCS	Pre-treatment	320, 273/47	51.8 ± 11.4	103/217	Medical therapy, surgery, TEVAR
Yuan, H *et al*., 2021 [34]	China	RCS	Pre-treatment	313, 264/49	48 ± 10	312/0	Emergency surgery
Zhou, Y *et al*., 2021 [17]	China	RCS	Pre-treatment	707, 552/155	46.6 ± 10.4	707/0	TAR+FET

Abbreviations: M, male; F, female; NR, not reported; RCS, retrospective cohort study; PCS, prospective cohort study; TEVAR, thoracic endovascular aortic repair; ICU MV, intensive care unit mechanical ventilation; TAR+FET, total aortic arch replacement combined with the frozen elephant trunk.

The quality evaluation results are shown in **Supplementary Table 4**, and the NOS score included in the study was 5–8 (total score was 9). Seven articles [27, 28, 29, 30, 31, 32, 34] were rated as medium-quality and three [17, 26, 33] as high-quality research.

### 3.2 Meta-Analysis Outcomes

**Fig. 2.**
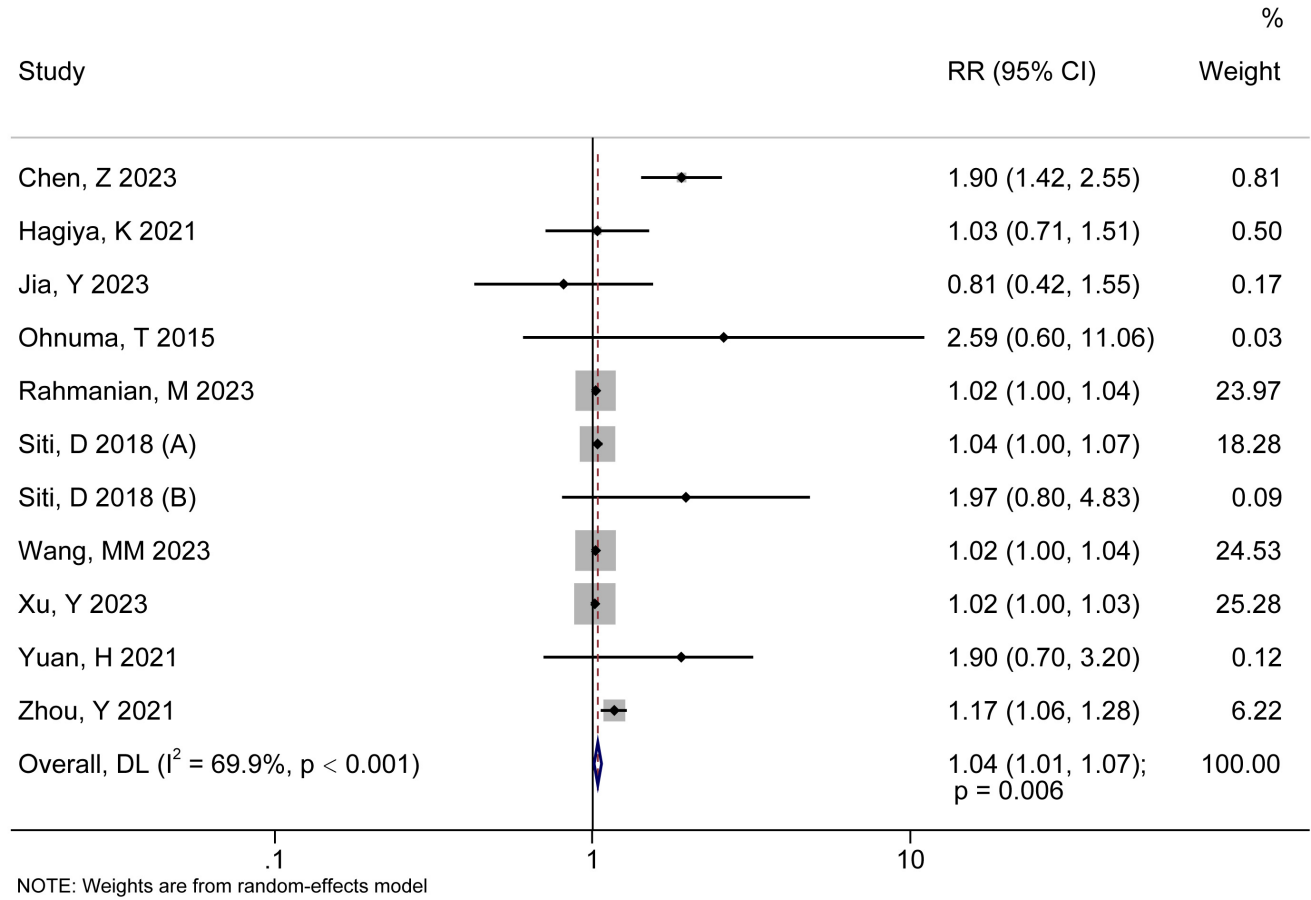
**The forest diagram of the correlation analysis between AAD death risk and HR**. RR, relative risk; DL, DerSimonian–Laird; 95% CI, 95% confidence interval.

The forest diagram of the correlation analysis between AAD mortality risk and HR was shown Fig. 2. The RR of the included studies was >1, excluding Jia *et al*. (2023) [30], which was <1, indicating a positive association between HR and AAD mortality risk. There was significant difference in statistical precision (I^2^ = 69.9, *p* < 0.001), and the combined results were RR (95% CI) = 1.04 (1.01, 1.07) (*p* = 0.006), which suggested that increased HR significantly increased AAD mortality risk.

### 3.3 Subgroup Analysis

**Fig. 3.**
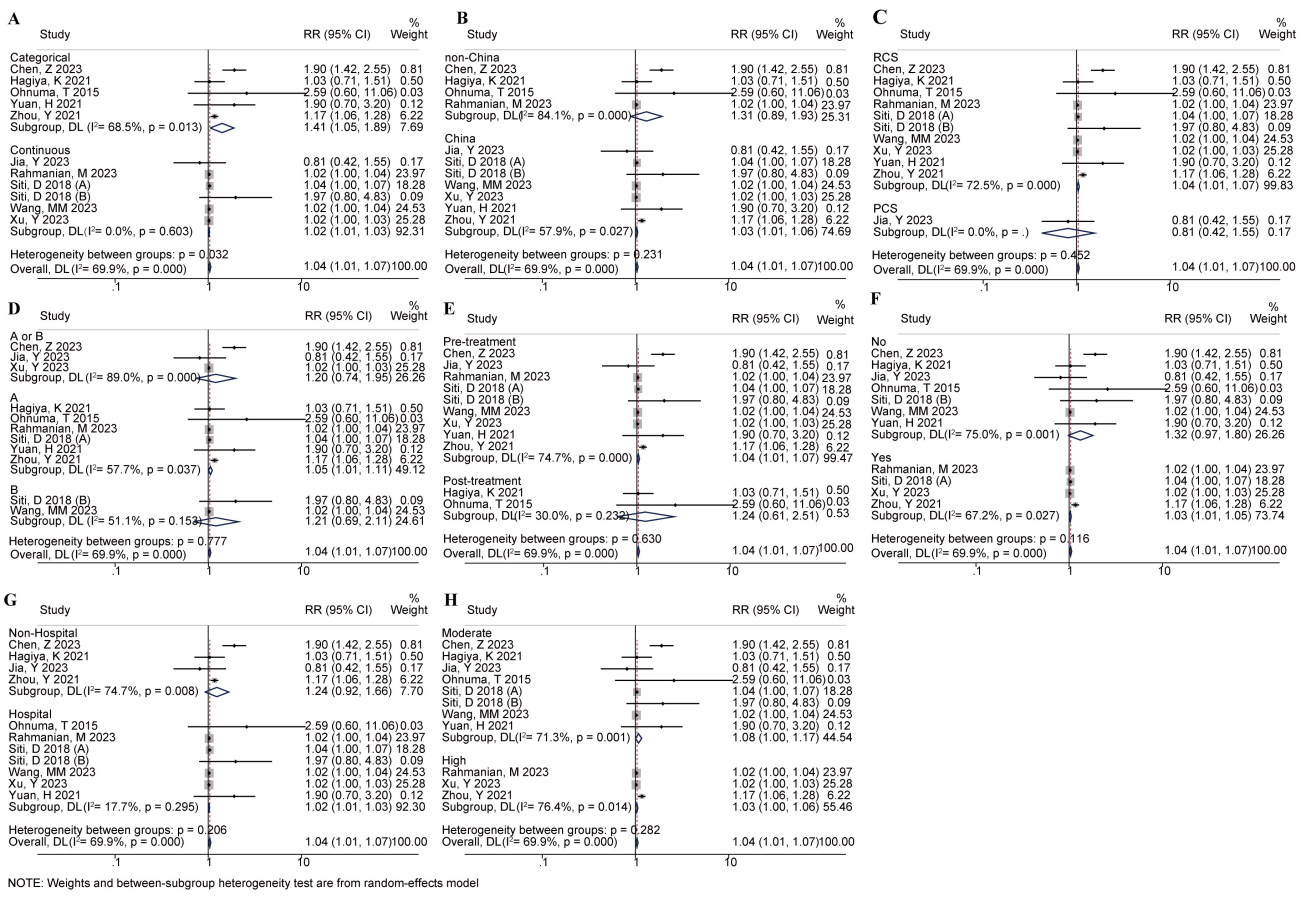
**Outcomes of subgroup analyses**. Classification subgroup (A), regional subgroup (B), study type subgroup (C), disease types subgroup (D), detection time subgroup (E), whether there are correction types subgroup (F), death type subgroup (G), and research quality subgroup (H).

Subgroup analysis was used to analyze the sources of heterogeneity. Fig. 3 and Table 2 showed the results of subgroup analysis. Whether as a categorical or continuous variable, the correlation between HR and AAD mortality risk was statistically significant, and the combined results were RR (95% CI) = 1.41 (1.05, 1.89) (*p* = 0.023) and RR (95% CI) = 1.02 (1.01, 1.03) (*p* < 0.001), respectively. Moreover, the combined results of China, RCS, Stanford type A of AAD, HR measurement before treatment, multivariate adjusted research, hospital mortality, and high-quality research were statistically significant (*p* < 0.05). The merging results of other subgroups were not significant. Excluding HR as a continuous variable, measured HR after treatment, and hospital mortality, there was no significant heterogeneity, and the heterogeneity of other subgroups was statistically significant. Therefore, the grouping variables were not the influencing factors of heterogeneity. Additionally, HR as a continuous or categorical variable showed a statistically significant difference between groups (*p* = 0.032).

**Table 2.** **Outcomes of subgroup analyses**.

Outcomes		No. of studies	RR (95% CI)	*p*_A_	Heterogeneity test	
					*p*	I^2^ (%)
Total		11	1.04 (1.01, 1.07)	0.006	<0.001	69.9
Comparison						
	Categorical	5	1.41 (1.05, 1.89)	0.023	0.013	68.5
	Continuous	6	1.02 (1.01, 1.03)	<0.001	0.603	0.0
Location						
	China	7	1.03 (1.01, 1.06)	0.014	0.027	57.9
	Non-China	4	1.31 (0.89, 1.93)	0.175	<0.001	84.1
Design						
	RCS	10	1.04 (1.01, 1.07)	0.006	<0.001	72.5
	PCS	1	0.81 (0.42, 1.55)	0.524	NA	NA
Stanford type						
	A	6	1.05 (1.01, 1.11)	0.029	0.037	57.7
	B	2	1.21 (0.69, 2.11)	0.510	0.153	51.1
	A or B	3	1.20 (0.74, 1.95)	0.453	<0.001	89.0
Detected time of HR						
	Pre-treatment	9	1.04 (1.01, 1.07)	0.007	<0.001	74.7
	Post-treatment	2	1.24 (0.61, 2.51)	0.559	0.232	30.0
Adjusted						
	No	7	1.32 (0.97, 1.80)	0.078	0.001	75.0
	Yes	4	1.03 (1.01, 1.05)	0.013	0.027	67.2
Mortality						
	Hospital	7	1.02 (1.01, 1.03)	<0.001	0.295	17.7
	Non-Hospital	4	1.24 (0.92, 1.66)	0.160	0.008	74.7
Quality						
	Moderate	8	1.08 (1.00, 1.17)	0.055	0.001	71.3
	High	3	1.03 (1.00, 1.06)	0.046	0.014	76.4

Abbreviations: *p*_A_, *p* value for test of the association.

### 3.4 Sensitivity Analysis

**Fig. 4.**
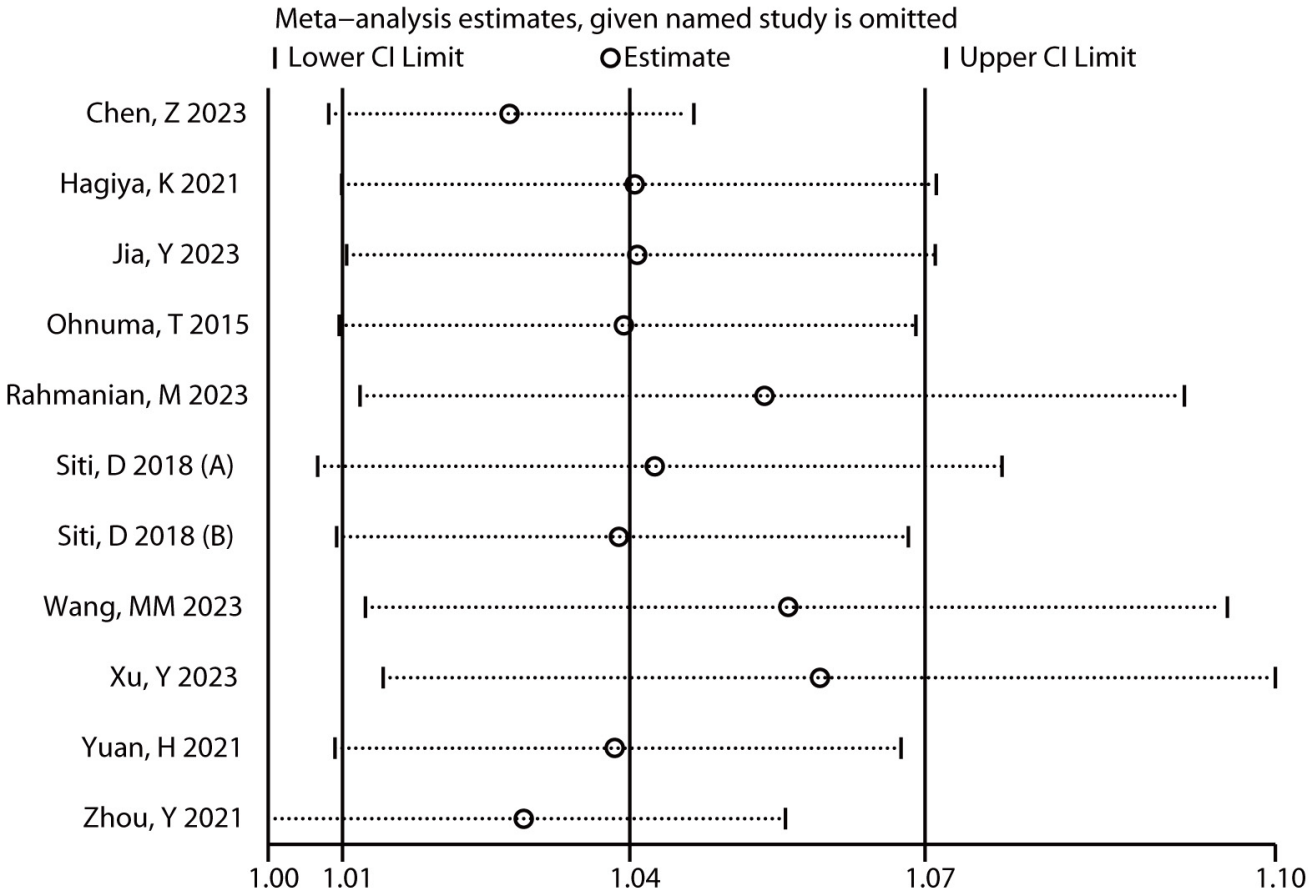
**The sensitivity analysis of the relationship between AAD death risk and HR**.

Sensitivity analysis showed that the range of the combined results had an RR (95% CI) = 1.03 (1.00, 1.05) to 1.06 (1.01, 1.10). Excluding any study, the combined results of the remaining studies remained statistically significant (*p* < 0.05) and stable (Fig. 4).

### 3.5 Publication Bias

**Fig. 5.**
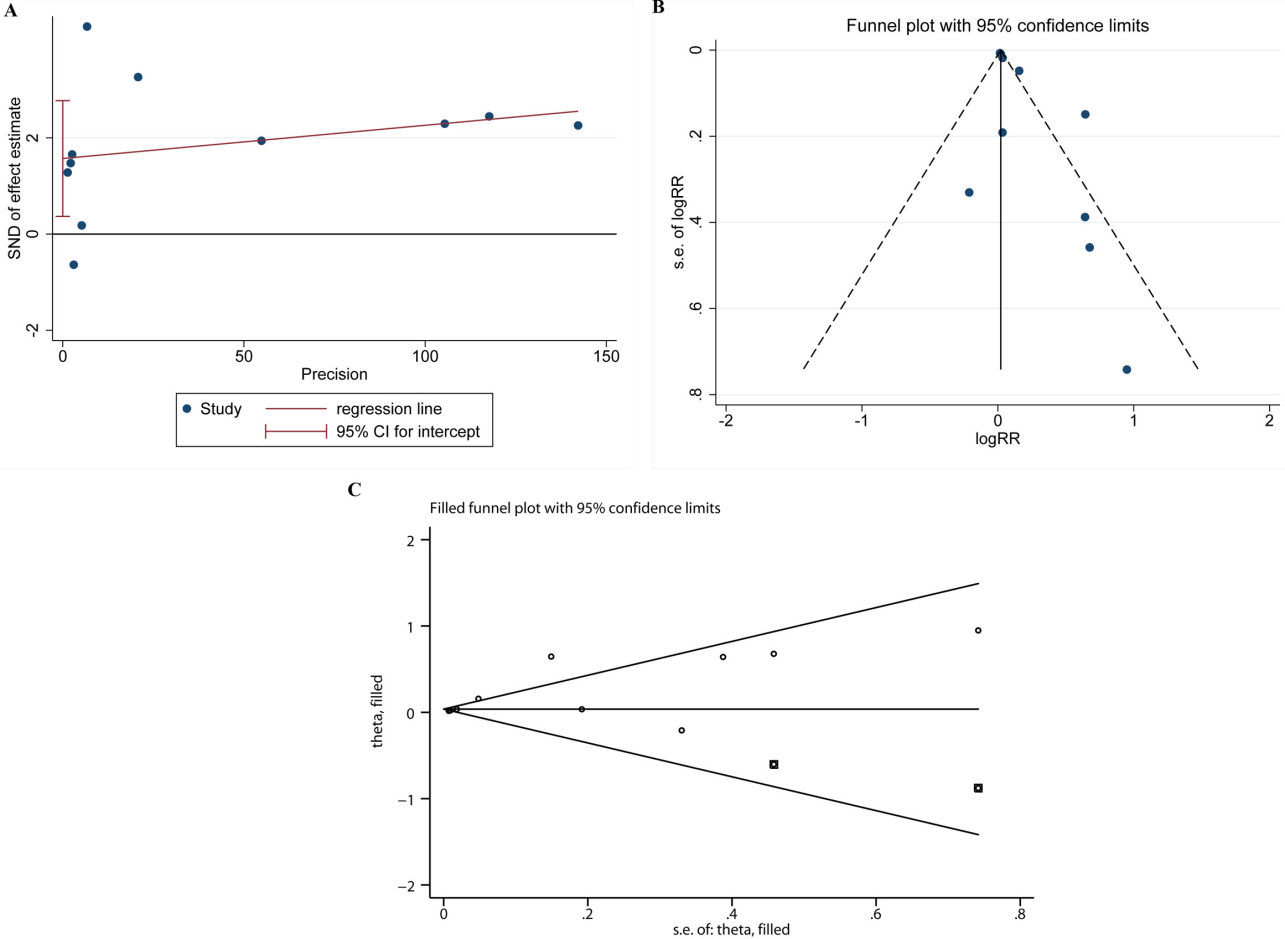
**The publication bias test**. Egger’s test (A), Egger’s funnel plot (B), and shear-fill method (C). SND, standard normal deviate.

The results of Egger’s test and funnel chart showed whether there was significant publication bias among studies. All major outcomes were significant by Egger test (*p* = 0.016) (Fig. 5A). The funnel chart revealed that the distribution symmetry of scatter plot was poor, suggesting an asymmetry in the studies reporting primary AAD results (Fig. 5B). Thus, shear-fill method was used to adjust the analysis. The combined result was RR (95% CI) = 1.038 (1.010, 1.066) (*p* = 0.008) after adding two filler studies (Fig. 5C), indicating that the combined result had little effect on publication bias.

## 4. Discussion

Recently, meta-analyses have focused on the surgical treatment scheme and prognosis of AAD [13, 35, 36]. Accurate early diagnosis and effective treatment of AAD remains of paramount importance to improve the survival rate of patients. Missed diagnosis of patients with AAD carries a significant mortality risk [20]. Our study showed that increased HR was significantly correlated with increased AAD mortality risk. However, the heterogeneity of the selected studies was significant regarding timing of HR monitoring, AAD type, and follow-up, which may affect the correlation strength and significance between HR and AAD. It is suggested that the follow-up study should form a unified standard for HR measurement time, grouping threshold, and study outcome evaluation to evaluate the relationship between them more accurately.

Currently, computed tomography (CT), magnetic resonance imaging (MRI), and transesophageal echocardiography (TEE) are usually performed to identify or exclude the AAD of patients, which are regarded as a grade I recommendation (evidence grade B) [37]. Besides the concerns caused by transferring potentially critical patients to radiology, the disadvantages of TEE, CT, and MRI mainly include the risk of venography and ionizing radiation. Consultation is not widely provided in many emergency departments [38]. Aside from imaging, biomarkers, such as D-dimer, troponins, and serum calcium, have been used to aid in AAD diagnosis. The potential problem of applying D-dimer in clinical practice lies in its poor specificity and lack of prospective verification of its application in decision-making [20, 39]. Other biomarkers, such as troponins [40] and serum calcium [41], exhibited the potential of risk stratification in the diagnosis of patients with AAD. However, it is expensive and inefficient for doctors and patients to use imaging techniques and biomarker detection to diagnose AAD, and not all hospitals are equipped with them. Therefore, a rapid, economical, and convenient diagnosis and prediction method is needed.

Resting HR is the core of cardiac output and is influenced by the changes in many diseases. Abnormal HR usually affects the amplitude and frequency of tensile stress on arterial wall and local hemodynamic environment, resulting in changes in endothelial cell structure and functions [42]. The imbalance of the autonomic nervous system, increased sympathetic nerve activity, and/or decreased parasympathetic nerve activity may be related to the pathogenesis of increased HR, increased blood pressure, diabetes, and obesity in some patients [43]. In the case of aortic coarctation, this autonomic imbalance may exacerbate tensile stresses on the arterial wall and changes in the local hemodynamic environment, thereby affecting endothelial cell structure and function. Current evidence shows that HR is an important indicator of cardiovascular diseases, including heart failure and AAD [44]. For example, Oliva *et al*. [45] expounded that HR can be used as a prognostic biomarker and is strongly associated with the prognosis of heart failure and acute heart failure. A retrospective study found that patients with sinus rhythm could significantly benefit from reduced HR. A large-scale meta-analysis (including 46 studies, including 1,246,203 patients) showed that increased HR was positively correlated with all-cause mortality and cardiovascular mortality, and the HR increased by 10 times/min. The overall all-cause mortality RR was 1.09 and 95% CI was 1.07–1.12, which showed that the mortality risk increased significantly. The RR of cardiovascular mortality was 1.08 and 95% CI was 1.06–1.10, indicating that the risk rate of cardiovascular mortality is obviously increased [46]. This suggests that HR control is critical for improving the prognosis of patients with cardiovascular diseases. Here, we demonstrated that HR is an influential independent risk factor for AAD. Chen *et al*. [28] found that HR (>90 bpm) was independently related to the high mortality of patients with severe AAD and had potential value in predicting the short- and long-term prognosis of patients with AAD. Hagiya *et al*. [29] suggested that patients with AAD after surgical treatment were more likely to have long-term aortic events in their HR at discharge ≥84 beats/min. These findings underscore the significance of HR control in preventing long-term complications in patients with aortic dissection. In the treatment of AAD, HR control may be a critical factor. Studies suggest that reducing HR can improve patient outcomes [17]. Patients benefit from reduced HR if it is controlled <80 bpm upon emergency department arrival. This study indicates a significant positive correlation between increased HR and AAD mortality risk. The association between HR and aortic dissection may operate through effects on autonomic balance, exacerbation of hemodynamic changes in the arterial wall, and impact on the prognosis of cardiovascular diseases. Therefore, HR control may be an important therapeutic strategy for improving the prognosis of patients with AAD.

Additionally, meta-subgroup analysis showed that China, RCS, Stanford type A, the study of measuring HR before treatment, the study after multi-factor correction, hospital mortality, and high-quality research had significant effects on heterogeneity (*p* < 0.05). The difference of regional heterogeneity may be that the detection of AAD in China was more sensitive than that in other regions. In the subgroup analysis of these variables, the between-group differences were not statistically significant (*p* > 0.05), indicating that none of these were a major cause of statistical precision. Contrastingly, HR, as a continuous or categorical variable, had a statistically significant between-group difference (*p* = 0.032), suggesting that it was a cause of statistical precision, thereby affecting the effect values. The sample size of our research was large. The heterogeneity test of statistical indicators improved the internal authenticity of the study.

Our study provided new insights into the risk management of AAD and helped to predict the diagnosis and progression in patients with AAD, thereby improving the survival rate of AAD. In 2010, the American Heart Association (AHA) guidelines [47] formally proposed that the initial treatment of Stanford type B AAD should reduce the stress of the aortic wall by controlling HR and blood pressure. However, further randomized controlled trials are needed to validate these results. This study laid a foundation for further research, particularly in determining that early detection and control of HR can help reduce AAD mortality. Our study was used to comprehensively evaluate the relationship between HR and AAD mortality risk, and subgroup analysis was used to evaluate the influence on the results. Although there was a significant publication bias, the clipping method results suggested that publication bias had little influence on the merger results. The one-by-one exclusion method demonstrated good stability of the meta-analysis.

This study has some limitations. The heterogeneity of included studies was significant, and subgroup analysis did not identify any significant influencing factors. Most of the included studies were retrospective, and there were many confounding factors, which may affect the authenticity of the results. Moreover, the included studies came from Asian countries, and more high-quality studies were needed to verify the extrapolation of the results. Therefore, more studies with a large sample, randomized and blind study design are required to explore the correlation between HR and AAD diagnosis to make the results more clinically significant.

## 5. Conclusions

This meta-analysis revealed a positive relationship between increased HR and increased mortality in patients with AAD. HR has convenient and excellent diagnostic performance in AAD diagnosis, and monitoring of HR may improve the prognosis of patients with AAD.

## Availability of Data and Materials

The datasets used in our study are available from the corresponding author on reasonable request.

## Supplementary Material

Supplementary material associated with this article can be found, in the online version, at https://doi.org/10.31083/RCM27755.
