# Supplementary material for: The Relationship Between Heart Rate and Mortality Risk in Patients With Acute Aortic Dissection: A Meta-Analysis
Source: Rev Cardiovasc Med. 2025 May 27;26(5):27755. doi: 10.31083/RCM27755 (PMC12135637; doi:10.31083/RCM27755)
Supplement: Supplementary file 1 [file 2153-8174-26-5-27755-s1.zip › Supplementary Tables/Supplementary Tables.docx]

**Supplementary Table 1 Search strategy of PubMed**

| Search | Query | Items found |
| --- | --- | --- |
| #1 | "aortic dissection"[MeSH Terms] OR "aortic dissection"[All Fields] OR "aortic dissections"[All Fields] | 28157 |
| #2 | "acute"[All Fields] OR "acutely"[All Fields] OR "acutes"[All Fields] | 1610966 |
| #3 | #1 AND #2 | 11344 |
| #4 | "heart rate"[MeSH Terms] OR "heart rate"[All Fields] | 268476 |
| #5 | #3 AND #4 | 88 |

**Supplementary Table 2 Search strategy of Embase**

| Search | Query | Items found |
| --- | --- | --- |
| #1 | acute:ti,ab | 1783773 |
| #2 | ('aortic dissection'/exp OR 'aortic dissection' OR 'aortic dissections') | 30997 |
| #3 | #1 AND #2 | 12164 |
| #4 | ('heart rate'/exp OR 'heart rate') | 320880 |
| #5 | #3 AND #4 | 346 |

**Supplementary Table 3 Search strategy of Web of Science**

| Search | Query | Items found |
| --- | --- | --- |
| #1 | Aortic Dissection OR Aortic Dissections (All Fields) | 21074 |
| #2 | Acute (All Fields) | 1236014 |
| #3 | #1 AND #2 | 8345 |
| #4 | "Heart rate" (All Fields) | 135820 |
| #5 | #3 AND #4 | 75 |

**Supplementary Table 4 Quality assessment of the cohort studies with Newcastle-Ottawa quality assessment scale**

| **Study** | **Representati-veness of the exposed cohort** | **Selection of the unexposed cohort** | **Ascertainment of exposure** | **Outcome of interest not present at start of study** | **Control for important factor or additional factor** | **Outcome assessment** | **Follow-up long enough for outcomes to occur** | **Adequacy of follow-up of cohorts** | **Total quality scores** |
| --- | --- | --- | --- | --- | --- | --- | --- | --- | --- |
| Chen, Z 2023 | -- | ☆ | ☆ | -- | -- | ☆ | ☆ | ☆ | 5 |
| Hagiya, K 2021 | ☆ | ☆ | ☆ | -- | -- | ☆ | ☆ | ☆ | 6 |
| Jia, Y 2023 | -- | ☆ | ☆ | ☆ | -- | ☆ | ☆ | ☆ | 6 |
| Ohnuma, T 2015 | -- | ☆ | ☆ | -- | -- | ☆ | ☆ | ☆ | 5 |
| Rahmanian, M 2023 | -- | ☆ | ☆ | -- | ☆☆ | ☆ | ☆ | ☆ | 7 |
| Siti, D 2018 | -- | ☆ | ☆ | -- | ☆ | ☆ | ☆ | ☆ | 6 |
| Wang, MM 2023 | -- | ☆ | ☆ | -- | -- | ☆ | ☆ | ☆ | 5 |
| Xu, Y 2023 | ☆ | ☆ | ☆ | -- | ☆☆ | ☆ | ☆ | -- | 7 |
| Yuan, H 2021 | ☆ | ☆ | ☆ | -- | -- | ☆ | ☆ | ☆ | 6 |
| Zhou, Y 2021 | ☆ | ☆ | ☆ | -- | ☆☆ | ☆ | ☆ | ☆ | 8 |
